# Robotic Local Fat Extirpation and Ureteric Reimplantation for Pelvic Lipomatosis with Ureteric Obstruction: Technical Considerations

**DOI:** 10.5152/tud.2022.22146

**Published:** 2022-09-01

**Authors:** Mallikarjuna Chiruvella, Ghouse Mohammed Syed, Shanti Darga, Purnachandra Reddy Kondakindi, Bhavatej Enganti, Rajesh Kumar Reddy Adapala

**Affiliations:** 1 Department of Urology, Asian Institute of Nephrology and Urology, Hyderabad, India

**Keywords:** Pelvic lipomatosis, hydroureteronephrosis, ureteric reimplantation, robotic surgery

## Abstract

**Background:**

Ureteric reimplantation is the treatment of choice for pelvic lipomatosis with ureteric obstruction. Pelvic adherent fat poses a technical challenge during this surgery.

**Description of Technique:**

We describe the robotic approach to facilitate the precise dissection of the ureter and bladder in adherent fat. After creating pneumoperitoneum and port placement, the ureter is exposed at the iliac crossing and dissected distally. Perivesical fat at the intended site of ureteric reimplantation is excised and cystotomy is done. Ureterovesical anastomosis is performed over a stent.

**Patients and Methods:**

Two patients with pelvic lipomatosis causing ureteric obstruction and renal function impairment underwent robotic ureteric reimplantation at our institute. Technical aspects and outcomes are discussed here.

**Results:**

Blood loss was minimal. No intra-operative or post-operative complication was noted. Renal function improved for both patients.

**Conclusion:**

Robotic approach helps to overcome the technical difficulties posed by adherent fat during ureteric reimplantation in pelvic lipomatosis.

## Background

Pelvic lipomatosis (PL) is characterized by excessive deposition of mature adipose tissue in the pelvis causing extrinsic compression of pelvic organs. Rarely it can cause ureteric obstruction leading to proximal hydroureteronephrosis (HUN) and renal failure. Chronic bladder wall irritation or even the compression of lymphatics may trigger the bladder mucosal proliferative processes causing cystitis cystica or glandularis. Excretory urography demonstrates the classical “teardrop or pear-shaped bladder,” besides HUN. Cross-sectional imaging by contrast-enhanced computed tomography or magnetic resonance imaging establishes the exact cause of obstruction by delineating fat deposition around the pelvic organs.^1^

Multiple management options were described in the literature for HUN and resultant renal failure in patients with PL. Results were suboptimal with conservative management strategies such as weight loss, prolonged antibiotics, and steroids. Surgical treatment options such as nephrostomy or simple cystectomy and ileal conduit were described, which negatively impact the quality of life. Hence, bladder sparing surgeries have gained more acceptance among the available modalities.^1,2^ Ge et al^2^ reported their experience of 8 patients who underwent a combination of pelvic fat extirpation and ureteral reimplantation. Among them, 4 underwent laparoscopic surgery in which 1 was converted to open due to severe pelvic fat adhesion. Tough adherent fat poses technical challenges during this surgery. Hence, Ge et al^2^ suggested to convert to open surgery in case of difficulty. With the advent of robotic technology, we have extended its utility to perform robot-assisted ureteric reimplantation to overcome the technical difficulties posed by adherent fat. Hereby we share our initial experience of 2 such cases and discuss the technical aspects.

## Description of the Technique

**Figure 1.**
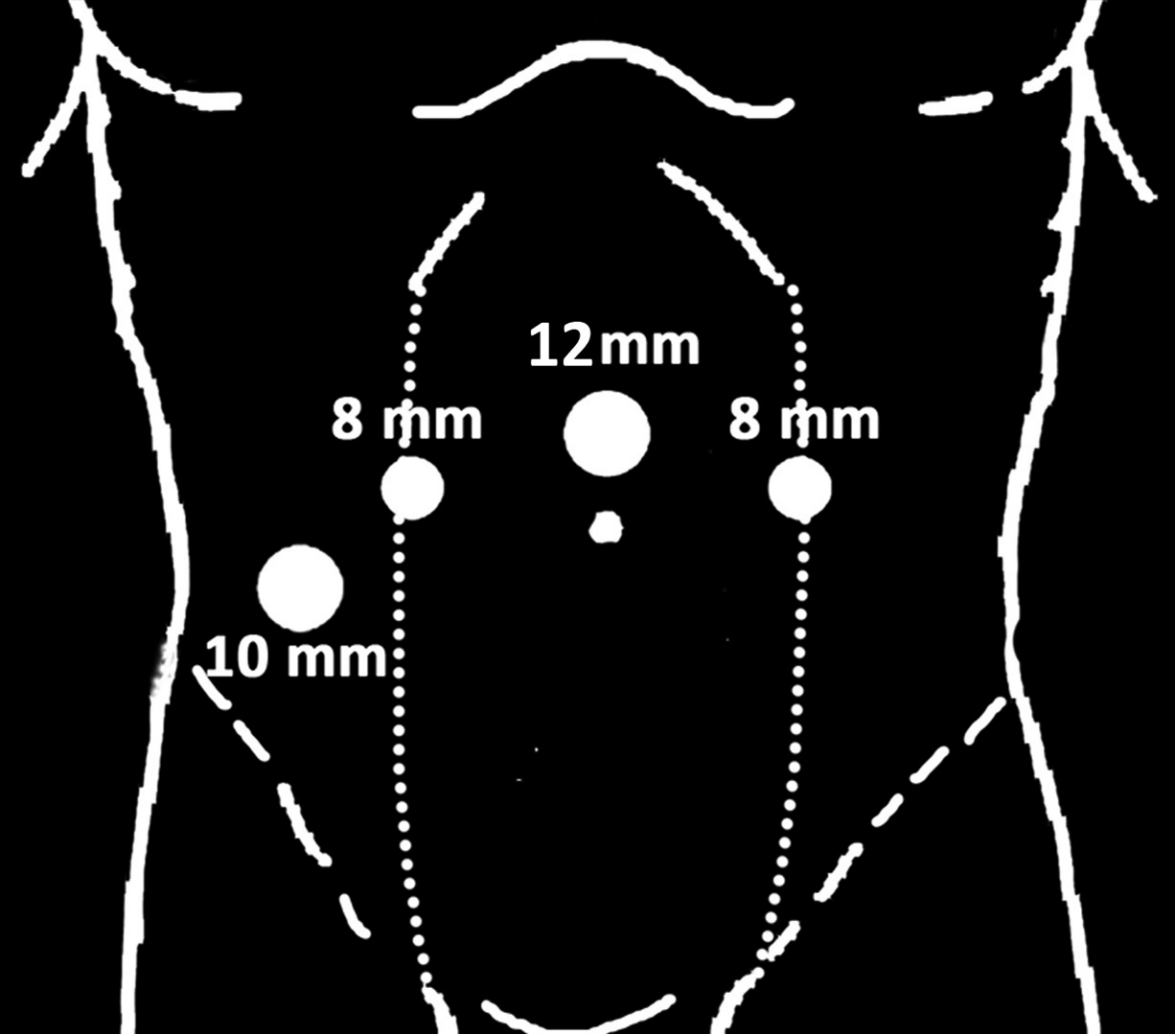
Port placement.

The patient is placed in a low lithotomy position. A large size per-urethral catheter is inserted. It facilitates intra-operative bladder distension and postoperative urine drainage. Pneumoperitoneum is created by Veress needle. A 12-mm camera port is placed just cephalad to the umbilicus in the midline. The peritoneal cavity is thoroughly assessed. In order to displace the bowel away from the pelvis, a steep Trendelenburg position is given. Two 8-mm robotic working ports are placed at the paramedian lines at the level of the umbilicus. These ports are located approximately 8-10 cm away from the camera port. A 10 mm assistant port is positioned at the flank area, around 3-5 cm above the iliac crest. This port can be placed ipsilaterally for unilateral ureteric reimplantation (Figure 1). The robot is docked. Monopolar curved scissors and fenestrated bipolar forceps are passed under vision.

**Figure 2.**
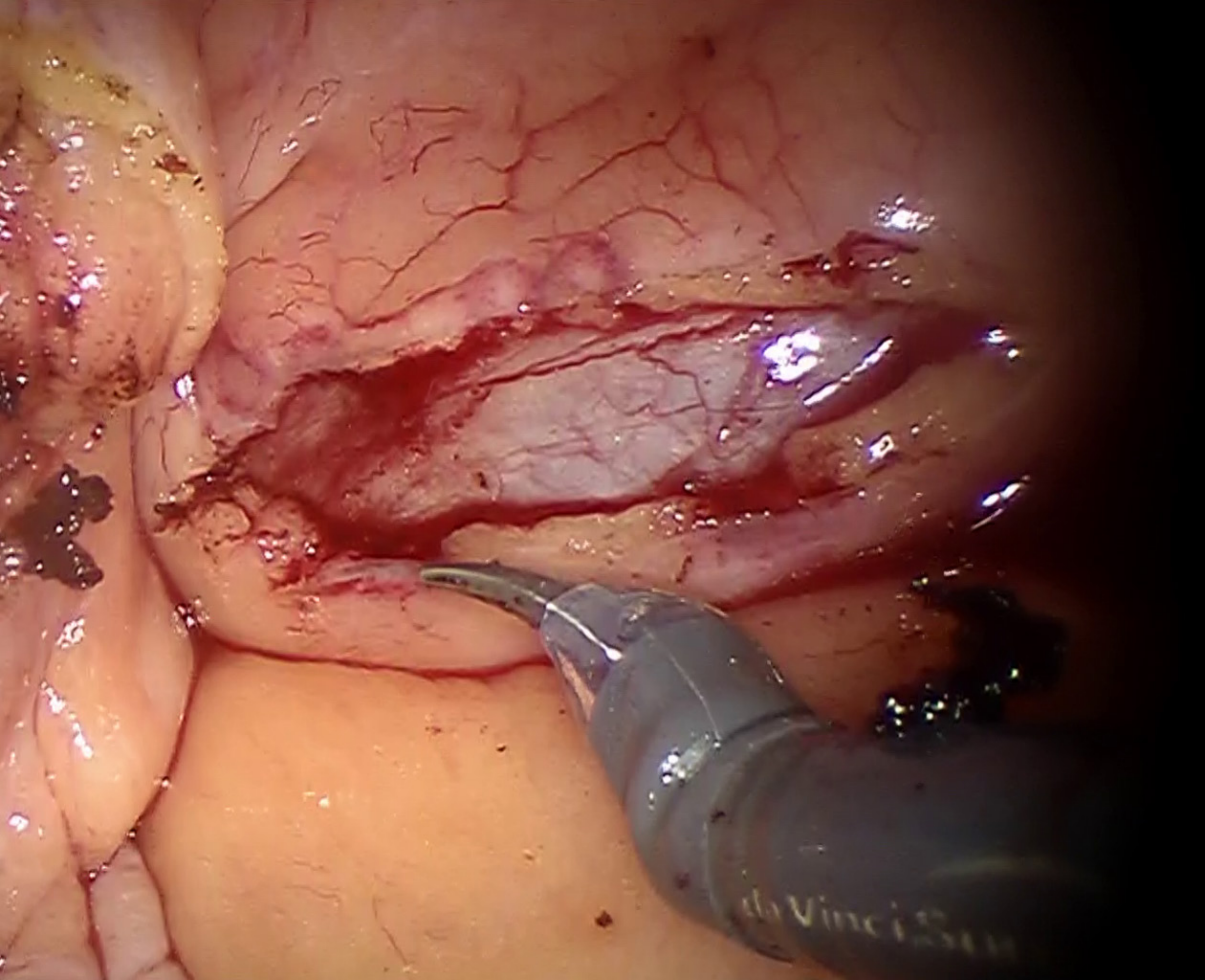
Peritoneal incision exposing the right ureter at the level of iliac crossing.

**Figure 3.**
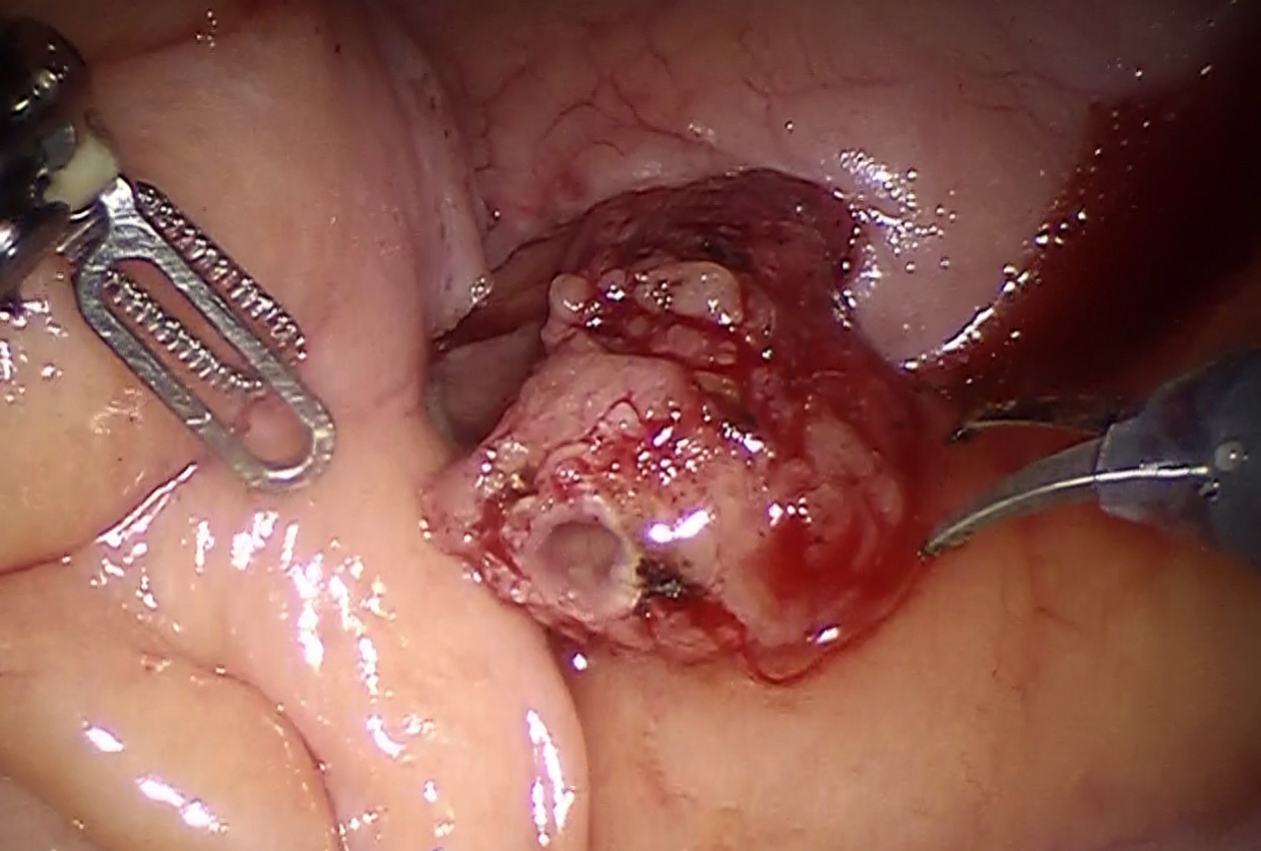
Transected distal ureteric end.

The ureter is exposed by incising the peritoneum at the level of iliac crossing (Figure 2) and then dissected distally as much as possible ensuring intact periureteric adventitia. This helps in subsequent tension-free anastomosis. Excessive and adherent perivesical fat makes this dissection difficult. The ureter is transected at this level (Figure 3). The urinary bladder is semi-distended with saline. Limited bladder drop is performed. The site of ureteric reimplantation at the bladder dome is determined.

**Figure 4.**
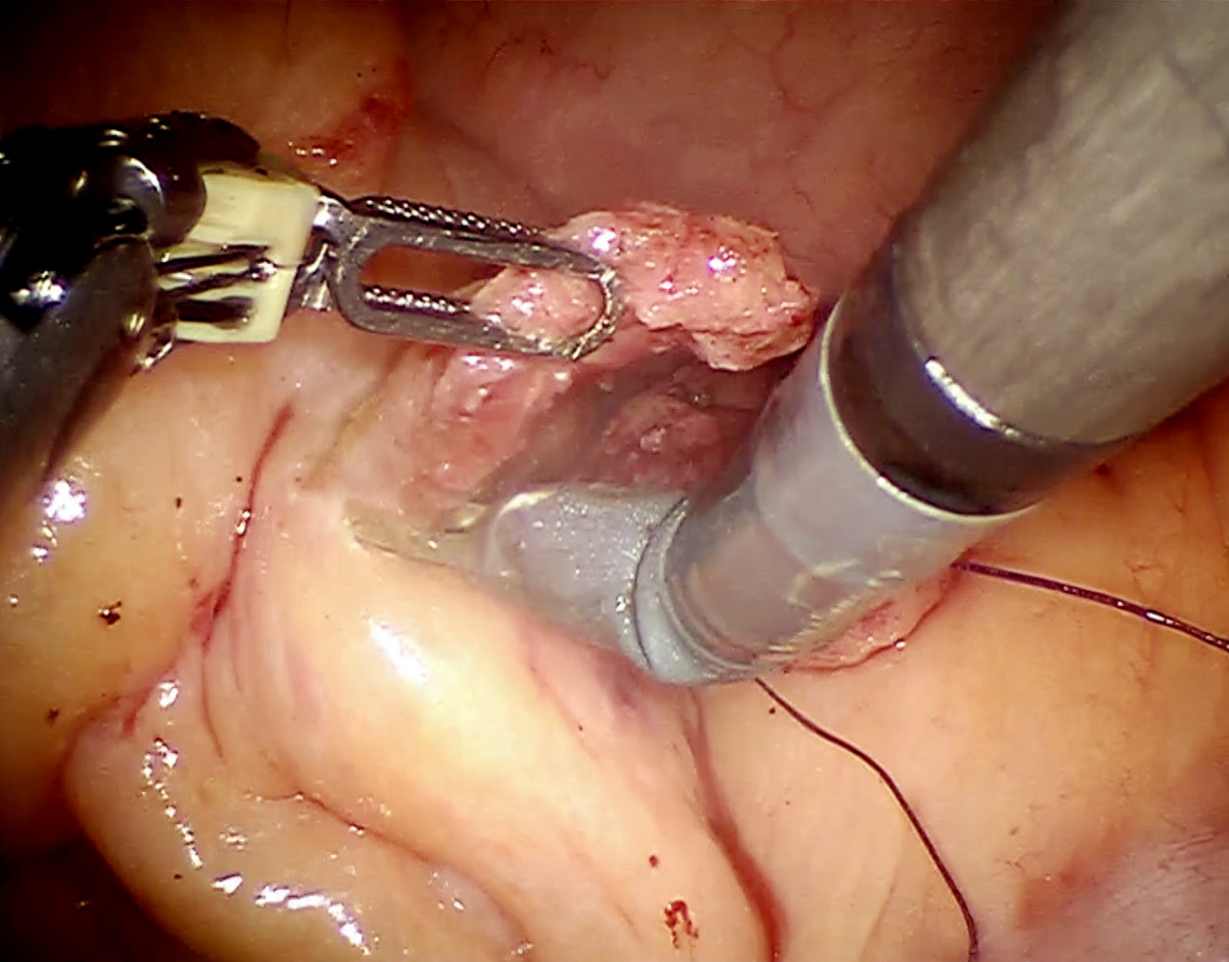
Local fat extirpation at the site of implantation over the bladder dome.

**Figure 5.**
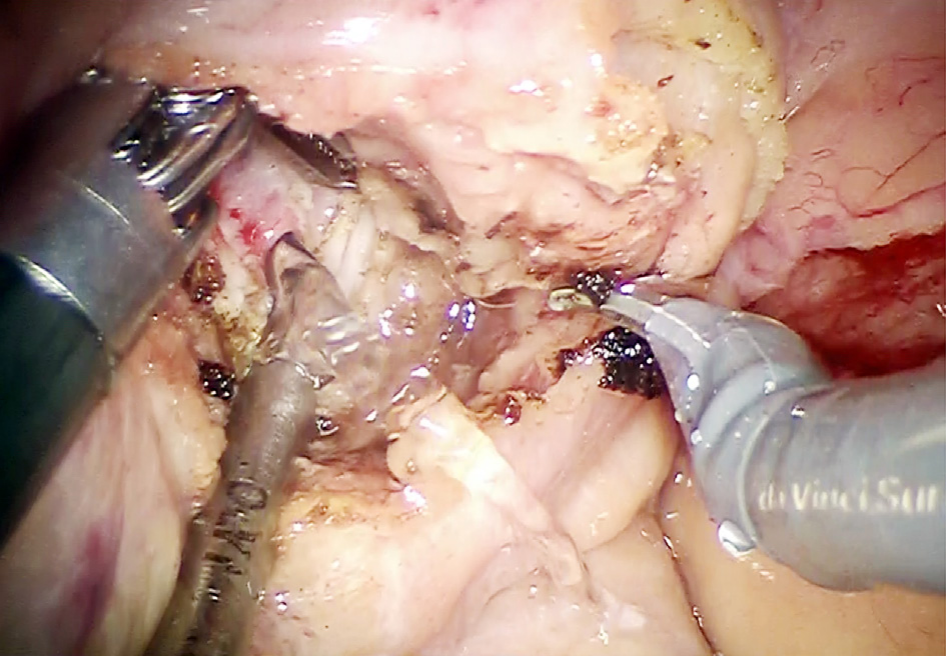
Cystotomy after local fat extirpation.

**Figure 6.**
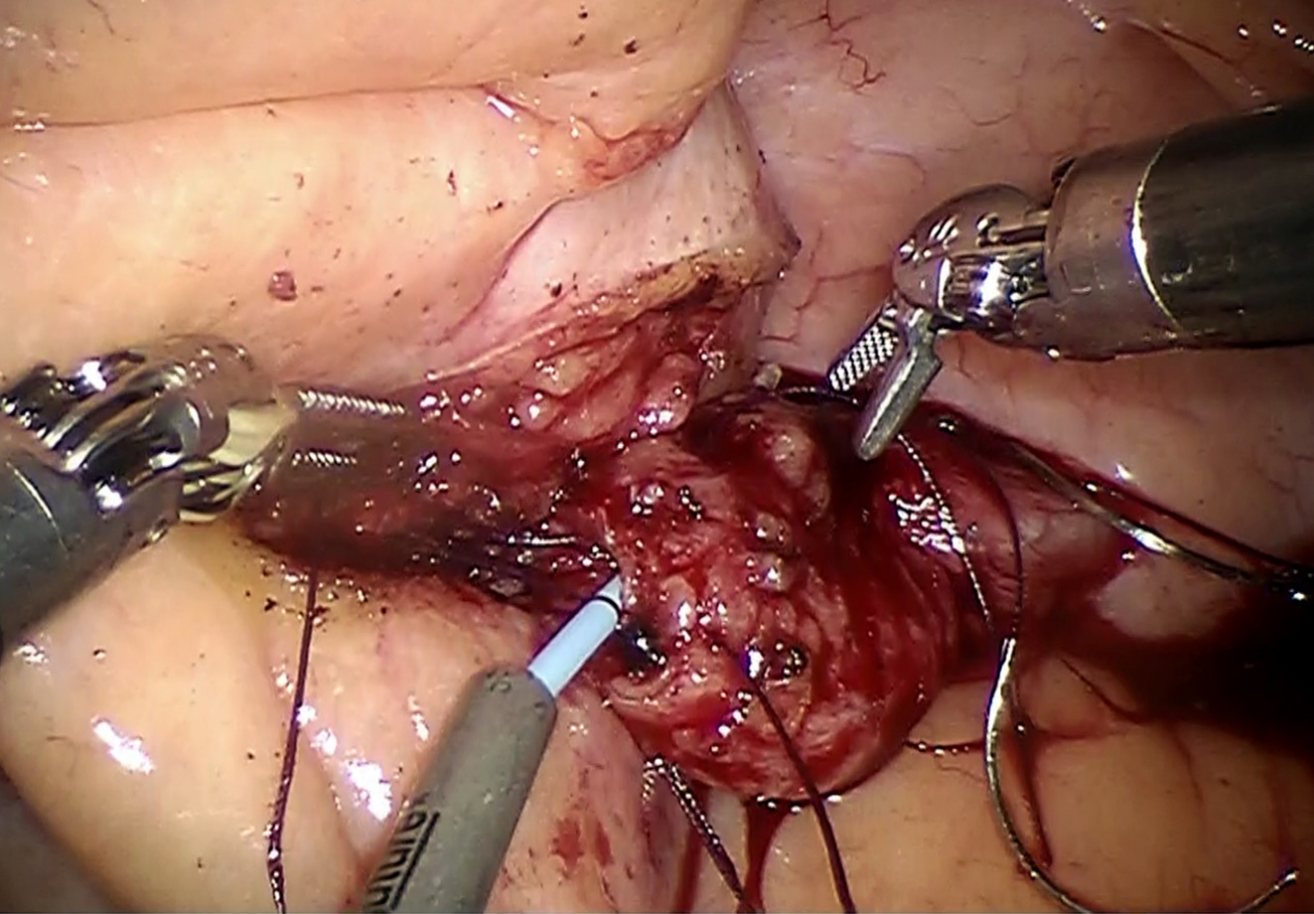
Vesico-ureteric anastomosis over a Double J stent.

Our preferred site of ureteric implantation is at the anterolateral aspect of the bladder dome. Adequate perivesical fat excision is performed to reach the bladder wall at the site of intended ureteric implantation (Figure 4). This specimen is sent for pathologic examination. A small, longitudinal cystotomy incision is made (Figure 5). A 4-0 absorbable suture is used for the anastomosis. Eversion mucosal sutures can be taken if the bladder mucosa is deeply buried. The lumen of the obstructed ureter in PL is usually wider. Hence, minimal or no spatulation of the ureteric end is required. Initial anchoring stitch is taken through the full-thickness bladder wall and ureteric wall at the 12 o’clock position. Interrupted, tensionless, extravesical, and ureterovesical anastomosis is performed. Once the medial half of the anastomosis is done, a 5-Fr DJ (Double J) stent is deployed (Figure 6). Once hemostasis is confirmed, the tube drain is secured and the robot is undocked.

Sanjay Prakash et al^3^ reported 5 cases who successfully underwent robotic perivesical fat extirpation and bilateral ureteral reimplantation. The major challenges during surgery in their series were lack of surgical planes in a fat-filled pelvis, need for adjustment of port positioning and redocking, difficulty in separating ureter from adherent fat, excessive bleeding due to hypervascular fat planes, and buried bladder mucosa making anastomosis difficult.

The robotic technique can overcome the technical challenges posed by conventional laparoscopy, especially with regard to the precision of dissection and suturing. This is possible due to “endo-wrist” technology featuring freedom of articulation. Improved visualization is another asset for robotic technology. These 2 factors also have helped in reducing the steep learning curve.^4,5^

## Patients and Methods

**Figure 7.**
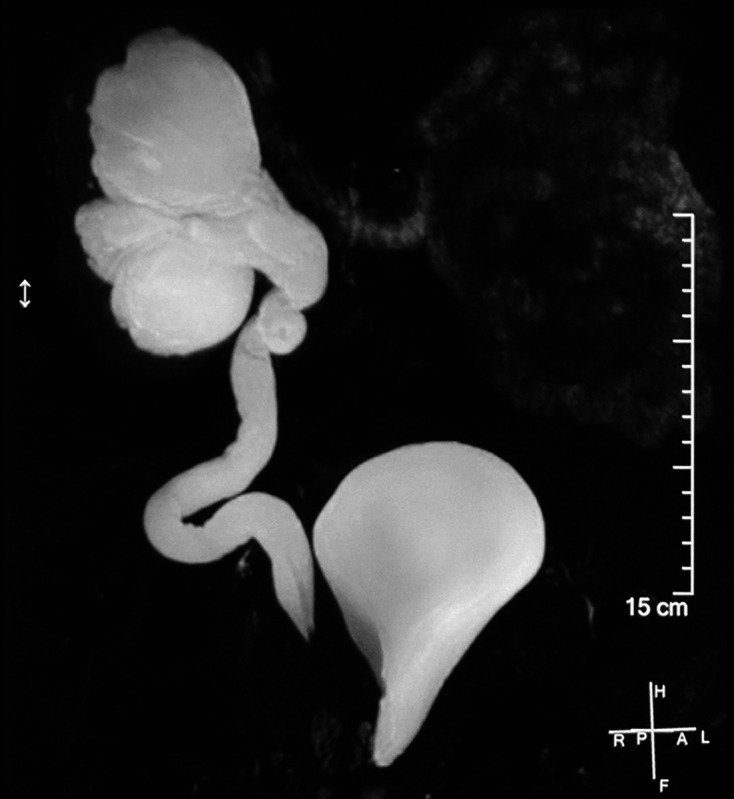
Contrast MRI showing pear-shaped bladder with right side solitary functioning kidney with gross HUN.

**Figure 8.**
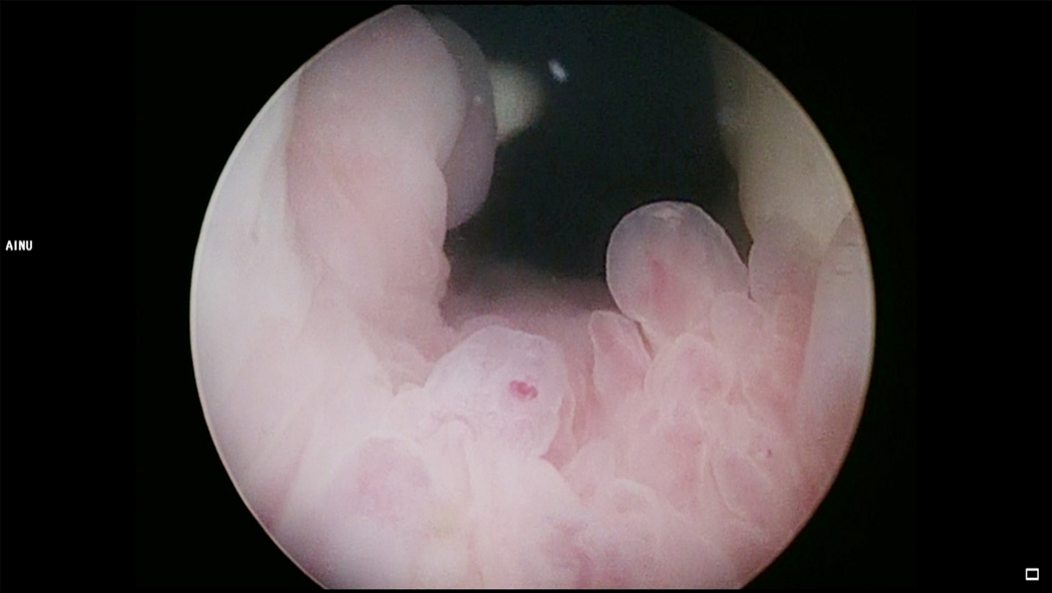
Cystoscopy- features of cystitis cystica.

**Case 1:** A 48-year-old gentleman underwent left nephrectomy elsewhere for a non-functioning kidney secondary to PL with ureteric obstruction. After 1 year, he presented to our hospital with altered renal parameters. Serum creatinine (Scr) was 3.15 mg/dL. Abdomen and pelvis MRI was suggestive of deposition of fatty tissue in the pelvis causing a pear-shaped bladder. Right-sided gross HUN due to distal ureteric compression was also noted (Figure 7). Cystoscopy showed an elevated bladder neck with the features of cystitis cystica (Figure 8). After stabilizing the renal function with initial DJ stenting, he underwent robotic ureteric reimplantation.

**Case 2:** A 37-year-old male was evaluated for raising Scr (1.8 mg/dL) and found to have bilateral HUN. Magnetic resonance imaging showed symmetric deposition of fat-density tissue in the pelvis with a pear-shaped bladder. Renogram study was suggestive of a significant delay in drainage of tracer bilaterally. He also underwent bilateral robotic ureteric reimplantation.

The da Vinci robotic Si surgical platform® (Intuitive Surgical, Sunnyvale, CA) was employed for both cases.

Ethical committee approval was received from the AINU Ethics Committee (Approval no: AINU 01/2019). Written informed consent was obtained from all participants who participated in this study.

## Results

No intra-operative complications were noted, blood loss was less than 50 mL and console time was less than 1 hour for both the patients. Intra-operatively dissection of the buried bladder wall and ureter in adherent pelvic fat was a major technical challenge, which could be successfully managed by a robotic approach.

The post-operative period was uneventful. Both patients were discharged on the third post-operative day and DJ stent removal was done after 6 weeks. Histopathological examination of the perivesical fat specimen showed benign mature adipose tissue with fibrosis. Serum creatinine level reduced to 2.0 mg/dL (nadir) for case 1 and it has been stable during 3 years follow-up period. Case 2 also has been on regular follow-up since 2 years and showing stable renal function.

## Conclusion

Ureteric reimplantation after fat extirpation in patients with PL causing HUN can stabilize the renal function. Excessive sticky fat makes the dissection difficult during this surgery. Hence, robotic approach appears to be much more feasible for this procedure as it is associated with better vision, flexibility, and precision with the added benefits of minimal invasive surgery.
